# Supplementary material for: Application of a dynamic optimization-based multi-attribute fusion method for fault detection
Source: PLoS One. 2025 Mar 17;20(3):e0311079. doi: 10.1371/journal.pone.0311079 (PMC11913290; doi:10.1371/journal.pone.0311079)
Supplement: Attachment — Submitted filename: PONE-D-24-05733_reviewer.pdf [file pone.0311079.s001.pdf]

PLOS ONE

Research on fracture identification methods

--Manuscript Draft--

**Manuscript Number:**	PONE-D-24-05733
**Article Type:**	Research Article
**Full Title:**	Research on fracture identification methods
**Short Title:**	Research and Application of a Dynamic Optimization-Based Multi-Attribute Fusion Method for Fault Detection
**Corresponding Author:**	Handong Huang<br>China University of Petroleum Beijing<br>CHINA
**Keywords:**	fault identification; strike-slip fault; attribute fusion; nonlinear smoothing; . fracture probability attribute
**Abstract:**	The Tarim Basin has seen a shift in the focus of oil and gas exploration, from interlayer karst to fracture-controlled karst. A significant oilfield, characterized by strike-slip faults, has been discovered in the Yueman area. However, identifying such fault zones is a challenging task due to the complex and chaotic seismic reflection characteristics, as well as the presence of seismic noise and other discontinuities. In order to improve oilfield production, accurate identification of strike-slip fault zones in ultra-deep dense limestone is a pressing issue. The seismic anomalies of such fault zones exhibit diverse characteristics, with the low-velocity zone of the fault causing a "beaded" reflection pattern. Traditional coherent and curvature attribute methods have large errors in identifying strike-slip faults, and they cannot adequately characterize the contour features of fracture-karst traps.To address these challenges, this study proposes a multi-attribute optimal surface-based fracture identification technology, based on forward simulation records. Seismic attributes that are sensitive to different types of strike-slip faults are selected, and multiple attributes are fused to obtain a fracture distribution map using the best surface voting algorithm. This method effectively suppresses noise that is irrelevant to fractures and is only sensitive to fracture information, allowing for the identification of subtle waveform changes caused by strike-slip faults. The accuracy and continuity of fracture identification are significantly improved. The proposed method has been successfully applied to the identification of strike-slip faults in the Yuemeng area of the Tarim Basin, yielding good results.
**Order of Authors:**	Chen Ma
	Handong Huang
	Youcai Tang
	Suo Cheng
	Chao Wang
	Xin Wang
**Additional Information:**	
**Question**	**Response**
**Financial Disclosure**<br><br>Enter a financial disclosure statement that describes the sources of funding for the work included in this submission. Review the submission guidelines for detailed requirements. View published research articles from *PLOS ONE* for specific	The author(s) received no specific funding for this work.

Powered by Editorial Manager® and ProduXion Manager® from Aries Systems Corporation

examples.<br><br>This statement is required for submission and **will appear in the published article** if the submission is accepted. Please make sure it is accurate.<br><br>**Funded studies**<br>Enter a statement with the following details:<br>• Initials of the authors who received each award<br>• Grant numbers awarded to each author<br>• The full name of each funder<br>• URL of each funder website<br>• Did the sponsors or funders play any role in the study design, data collection and analysis, decision to publish, or preparation of the manuscript?<br><br>Did you receive funding for this work?	
**Competing Interests**<br><br>Use the instructions below to enter a competing interest statement for this submission. On behalf of all authors, disclose any competing interests that could be perceived to bias this work—acknowledging all financial support and any other relevant financial or non-financial competing interests.<br><br>This statement is required for submission and **will appear in the published article** if the submission is accepted. Please make sure it is accurate and that any funding sources listed in your Funding Information later in the submission form are also declared in your Financial Disclosure statement.<br><br>View published research articles from *PLOS ONE* for specific examples.	The authors have declared that no competing interests exist.

**NO authors have competing interests**<br><br>Enter: *The authors have declared that no competing interests exist.*<br><br>**Authors with competing interests**<br><br>Enter competing interest details beginning with this statement:<br><br>*I have read the journal's policy and the authors of this manuscript have the following competing interests: [insert competing interests here]*<br><br>* typeset	
**Ethics Statement**<br><br>Enter an ethics statement for this submission. This statement is required if the study involved:<br><br>• Human participants<br>• Human specimens or tissue<br>• Vertebrate animals or cephalopods<br>• Vertebrate embryos or tissues<br>• Field research<br><br>Write "N/A" if the submission does not require an ethics statement.<br><br>General guidance is provided below. Consult the submission guidelines for detailed instructions. **Make sure that all information entered here is included in the Methods section of the manuscript.**	N/A

Powered by Editorial Manager® and ProduXion Manager® from Aries Systems Corporation

**Format for specific study types**<br><br>**Human Subject Research (involving human participants and/or tissue)**<br>• Give the name of the institutional review board or ethics committee that approved the study<br>• Include the approval number and/or a statement indicating approval of this research<br>• Indicate the form of consent obtained (written/oral) or the reason that consent was not obtained (e.g. the data were analyzed anonymously)<br><br>**Animal Research (involving vertebrate animals, embryos or tissues)**<br>• Provide the name of the Institutional Animal Care and Use Committee (IACUC) or other relevant ethics board that reviewed the study protocol, and indicate whether they approved this research or granted a formal waiver of ethical approval<br>• Include an approval number if one was obtained<br>• If the study involved *non-human primates*, add *additional details* about animal welfare and steps taken to ameliorate suffering<br>• If anesthesia, euthanasia, or any kind of animal sacrifice is part of the study, include briefly which substances and/or methods were applied<br><br>**Field Research**<br><br>Include the following details if this study involves the collection of plant, animal, or other materials from a natural setting:<br>• Field permit number<br>• Name of the institution or relevant body that granted permission	
**Data Availability**<br><br>Authors are required to make all data underlying the findings described fully available, without restriction, and from the time of publication. PLOS allows rare exceptions to address legal and ethical concerns. See the PLOS Data Policy and FAQ for detailed information.	Yes - all data are fully available without restriction

Powered by Editorial Manager® and ProduXion Manager® from Aries Systems Corporation

A Data Availability Statement describing where the data can be found is required at submission. Your answers to this question constitute the Data Availability Statement and **will be published in the article**, if accepted.<br><br>**Important:** Stating 'data available on request from the author' is not sufficient. If your data are only available upon request, select 'No' for the first question and explain your exceptional situation in the text box.<br><br>Do the authors confirm that all data underlying the findings described in their manuscript are fully available without restriction?	
**Describe where the data may be found in full sentences. If you are copying our sample text, replace any instances of XXX with the appropriate details.**<br><br>• If the data are **held or will be held in a public repository**, include URLs, accession numbers or DOIs. If this information will only be available after acceptance, indicate this by ticking the box below. For example: *All XXX files are available from the XXX database (accession number(s) XXX, XXX.).*<br>• If the data are all contained **within the manuscript and/or Supporting Information files**, enter the following: *All relevant data are within the manuscript and its Supporting Information files.*<br>• If neither of these applies but you are able to provide **details of access elsewhere**, with or without limitations, please do so. For example:<br><br>*Data cannot be shared publicly because of [XXX]. Data are available from the XXX Institutional Data Access / Ethics Committee (contact via XXX) for researchers who meet the criteria for access to confidential data.*<br><br>*The data underlying the results presented in the study are available from (include the name of the third party*	the data are owned by a third party and authors do not have permission to share the data

and contact information or URL).<br>• This text is appropriate if the data are owned by a third party and authors do not have permission to share the data.<br><br>* typeset	
Additional data availability information:	

Click here to access/download;Manuscript;Fault Detection.pdf

Research and Application of a Dynamic Optimization-Based Multi-Attribute Fusion Method for Fault Detection

Chen Ma [1,†], Handong Huang [1,†,*], Youcai Tang [1,*] Suo Cheng [2] Chao Wang [2] and Xin Wang [2]

[1] State Key Laboratory of Petroleum Resources and Prospecting, College of Geophysics, China University of Petroleum, Beijing 102249, China
[2] Exploration and Development Research Institute, PetroChina Tarim Oilfield Branch, Korla,84100,China
* Correspondence: webhhd@163.com (H.H.) tangyc@cup.edu.cn(Y.T.)
† Collage of Geophysics, China University of Petroleum, Fuxue Road, Changping District, Beijing, China.

Abstract: The Tarim Basin has seen a shift in the focus of oil and gas exploration, from interlayer karst to fracture-controlled karst. A significant oilfield, characterized by strike-slip faults, has been discovered in the Yueman area. However, identifying such fault zones is a challenging task due to the complex and chaotic seismic reflection characteristics, as well as the presence of seismic noise and other discontinuities. In order to improve oilfield production, accurate identification of strike-slip fault zones in ultra-deep dense limestone is a pressing issue. The seismic anomalies of such fault zones exhibit diverse characteristics, with the low-velocity zone of the fault causing a "beaded" reflection pattern. Traditional coherent and curvature attribute methods have large errors in identifying strike-slip faults, and they cannot adequately characterize the contour features of fracture-karst traps.To address these challenges, this study proposes a multi-attribute optimal surface-based fracture identification technology, based on forward simulation records. Seismic attributes that are sensitive to different types of strike-slip faults are selected, and multiple attributes are fused to obtain a fracture distribution map using the best surface voting algorithm. This method effectively suppresses noise that is irrelevant to fractures and is only sensitive to fracture information, allowing for the identification of subtle waveform changes caused by strike-slip faults. The accuracy and continuity of fracture identification are significantly improved. The proposed method has been successfully applied to the identification of strike-slip faults in the Yuemeng area of the Tarim Basin, yielding good results.

Keywords: fault identification; strike-slip fault; attribute fusion; nonlinear smoothing;. fracture probability attribute

1. Introduction

The deep-seated strike-slip faults in western China play a pivotal role in controlling substantial reserves of oil and gas. Both the main fault zone and secondary faults along the strike-slip fault exhibit enhanced displays of oil and gas, demonstrating robust fault-controlled storage and enrichment characteristics. Nevertheless, exploring ultra-deep fault-controlled carbonate reservoirs proves to be exceedingly complex, particularly within the strike-slip fault fracture zone where reservoirs exhibit significant heterogeneity. Reservoir characteristics suggest that storage spaces are predominantly influenced by dissolution or tectonic factors. Therefore, the identification of the strike-slip fault zone emerges as a critical challenge in seismic exploration. he burial depth of Ordovician oil and gas reservoirs in western China ranges from 7500 to 9000 meters, placing them in the ultra-deep category. Additionally, the seismic data quality for the target layer section is suboptimal. On seismic profiles, the fracture and broken zones of Ordovician carbonate reservoirs exhibit varying energy and chaotic reflection characteristics. These features are distributed in a strip shape on the plane[1,2]. In the exploration of Ordovician carbonate fractured reservoirs in western China for oil and gas, seismic reflections characterized by intense

energy 'beads' are predominantly aligned with high-yield oil and gas wells[3], While strip-shaped chaotic seismic reflections are indicative of high-quality reservoir characteristics, existing technologies excel in accurately identifying strong 'beads' reflections on seismic profiles. However, direct identification of strip-shaped chaotic reflections poses a challenge.Currently, various seismic attributes are employed in fault detection, encompassing measures such as the similarity [4] and coherence [5,6] of seismic reflection continuity, as well as the analysis of variance [7],curvature [8,9], and gradient amplitude [10] associated with reflection discontinuity.

These attributes exhibit certain efficacy in identifying primary strike-slip faults but are less effective for secondary ones. Despite leveraging seismic response characteristics of strike-slip faults, employing methods such as root mean square amplitude, amplitude gradient, multi-data fusion prediction [11], and structural tensor [12] for strike-slip fault identification remains limited to effectively distinguishing strong 'beads'-shaped reflections, with less clarity in identifying strip-shaped chaotic reflection characteristics.To enhance fault features and distinguish faults from other seismic image features, a commonly used approach involves utilizing a vertically stretched window during seismic coherence calculations. This method assumes that faults are more vertically oriented than other geological features, suppressing the latter. However, faults are rarely strictly vertical and may not necessarily be perpendicular to the seismic reflection surface. Pedersen et al. [14] proposed an ant tracking method that enhances fault features along 'artificial ant' paths, assuming these paths correspond to faults. Alternative approaches suggested by other scholars [15,16] involve smoothing along fault trends and dips to enhance fault features by exploring various combinations of fault trends and dips. Hale [17] calculates fault possibility or fault orientation similarity by smoothing both the numerator and denominator of similarity along fault trends and dips. Nevertheless, these methods often yield multiple solutions and are susceptible to illusions that do not align with structural shadows.

Therefore, this paper discusses the fusion of multiple preferred attributes for attribute fusion data volume using the optimal surface voting algorithm [18] to solve global maximization optimal paths and surfaces for multi-attribute fusion data volumes. Compared with traditional methods, this method fuses multiple preferred attributes to break through the limitations of single attributes and improve the accuracy of strike-slip fault interpretation. At the same time, using the optimal surface voting algorithm can calculate smooth paths or surfaces from high-noise or discontinuous features to improve fracture characterization accuracy and continuity. The application shows that this method can effectively identify strong energy "beads" shaped reflections and chaotic seismic reflection characteristics with obvious effects on identifying strike-slip faults.

2. Technical Approach

This paper integrates the developmental characteristics of faults in the research area with prior geological insights to establish geological models of strike-slip faults at different scales. Forward simulations are then conducted to serve as a foundation for attribute optimization in strike-slip fault identification. Based on the forward data volume, diverse seismic attributes are calculated and optimized. Multiple seismic attributes with the most effective identification outcomes are selected, and the attribute ratio fusion method is employed for a comprehensive evaluation of strike-slip faults.

Subsequently, utilizing the attribute fusion data volume, the dynamic path optimization algorithm is employed for precise fault characterization. The results of the path optimization calculations are combined with the attribute fusion data volume to compute the fault probability attribute. This not only enhances the accuracy of strike-slip fault identification but also improves the continuity of fault characterization. Finally, through a comparison of the model fault distribution with the fault identification results presented in this paper, the accuracy of the proposed method is verified. The method is further applied to actual seismic data, demonstrating its practical utility.

2.1. *Attribute Optimization Based on Forward Model*

2.1.1. Establishment of Forward Model

The pattern of strike-slip faults exhibits complexity, and seismic anomalies manifest in diverse forms. To assess the efficacy of this method in a three-dimensional data volume, this paper employs a three-dimensional strike-slip fault geological model (Figure 1a) for forward simulation. Simulated seismic records are obtained through wave equation forward modeling, utilizing a real velocity model. Subsequent to reverse time migration processing, post-stack Laplace filtering, and FK domain filtering, post-stack seismic data (Figure 1b) is generated. The seismic data has a sampling interval of 6m and a trace spacing of 12.5m.Building upon this foundation, the paper undertakes fracture identification using multiple attributes. Attributes are optimized based on identification effectiveness, thereby providing a foundation for subsequent research endeavors.

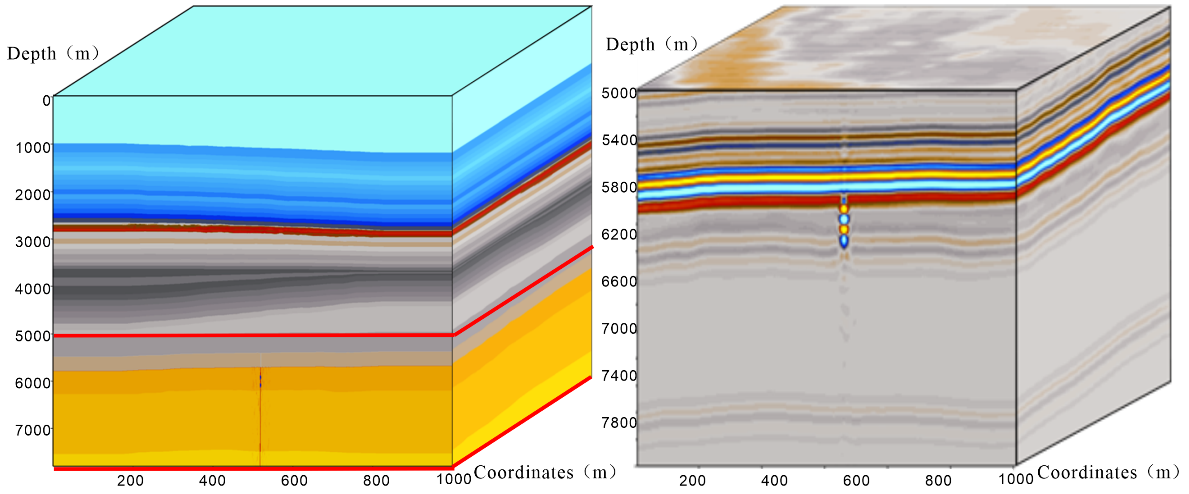

Figure 1. Comparison between forward model data volume and forward geological model a Geological model b Reverse time migration data volume

2.1.2. Fracture Attribute Analysis

Seismic data encompasses various types, each with distinct advantages and limitations in detecting seismic faults. The seismic anomalies within strike-slip fault zones are diverse, posing a challenge for accurate characterization using a single attribute. The adoption of a multi-attribute fusion method proves effective in enhancing the accuracy and reliability of seismic fault identification.This paper employs a multi-attribute fusion approach to comprehensively analyze different types of data, aiming for more precise determination of key information such as the location, shape, and nature of seismic faults. In the attribute fusion process, different seismic attributes undergo screening. By optimizing attributes, the most representative and predictive attributes are selected, and irrelevant or redundant attributes are eliminated to reduce complexity.Attribute optimization serves a dual purpose: it reduces computational costs, accelerates computation, and improves data processing efficiency, while also enhancing the accuracy and reliability of attribute fusion data, thereby improving prediction accuracy. This underscores the crucial role of attribute optimization as a key component influencing the overall performance and effectiveness of the model.

Commonly employed attributes in fracture identification include coherence attributes, curvature attributes, amplitude attributes, and amplitude gradient tensor attributes. The paper investigates the seismic response characteristics in different reservoir configurations based on typical fracture-controlled carbonate rock models and forward data. Distinct seismic attributes and characterization techniques are applied to various configuration units. The resulting seismic attributes are compared and optimized. The optimized seismic attributes undergo attribute fusion, contributing to the refinement of the accuracy and reliability of strike-slip fault characterization.

(1) Coherence Attribute There are currently three algorithms for coherence: The first generation coherence has the fastest calculation speed but its noise resistance is the worst; it is not suitable for low signal-to-noise ratio data in deep layers in research areas; The second generation coherence algorithm is slightly slower than the first generation coherence algorithm but its noise resistance has improved; it is more suitable for large work areas with low signal-to-noise ratio; The third generation coherence or eigenvalue coherence algorithm can eliminate factors such as multipath and lateral scattering that affect coherence; it improves resolution and clarity but is sensitive to seismic noise and interference; it requires high data quality.

(2) Curvature Attribute Curvature is a two-dimensional attribute of a curve; it is an inverse radius of a circle; curvature size can reflect how curved an arc is; larger curvature means more curvature. For brittle rocks, fracture development is proportional to curvature; therefore structural curvature can be used to predict fracture development. Structural curvature in three-dimensional seismic interpretation is calculated based on layer position; it reflects how curved any point on an interpreted layer position is.

(3) Gradient Structure Tensor Attribute A karst body is a combination of caves, karst holes, and fissures; conventional fracture detection attributes such as fine coherence or ant bodies mainly characterize internal fracture distribution features within karst bodies; they cannot well characterize outline features within karst body closures. Gradient structure tensor is a mathematical tool used to describe local structural features in images or signals. In processing seismic images, local structural features can be extracted by calculating gradient vectors and structure tensors. By calculating principal axis direction and size from gradient tensors we can determine potential fault locations and directions in images thus assisting with identifying and locating seismic faults. This method can improve accuracy and efficiency in processing seismic images; it has important application value for seismic exploration.

(4) Amplitude Attribute Amplitude attribute is one of the most basic attributes in seismic waveforms; it refers to amplitude size in seismic waveforms. Seismic faults usually cause phenomena such as reflection or refraction when waves propagate underground thus forming different amplitude sizes in waveforms. By analyzing changes in waveform amplitude, we can extract essential information, such as the location or shape of seismic faults. Processing amplitude attributes enhances the accuracy and reliability of seismic fault identification. In our model, we utilize coherence, curvature, amplitude, and gradient structure tensor attributes for fracture identification (refer to Figure 2). The presence of a low-speed zone caused by fractures leads to bead-like reflections, resulting in errors in traditional coherence and curvature attribute identification of strike-slip fractures. A comparison between the coherence attribute profile (Figure 2c) and the forward model profile (Figure 2a) reveals poor coherence on both sides of the bead-like reflections, indicating a mismatch with the fracture location. Similarly, comparing the curvature attribute profile (Figure 2d) with the forward model profile (Figure 2a) shows an increase in curvature on both sides, causing a significant error in identifying the location of strike-slip fractures.

The gradient structure tensor attribute (Figure 2e), when compared with the forward model profile (Figure 2a), accurately characterizes the outline of the karst body, highlighting high-value areas consistent with reflection anomalies within the karst body. This observation reflects the comprehensive influence from various elements in the karst body configuration.

Significant bead-like reflections within strike-slip fractures are evident in the amplitude attribute (Figure 2f). While the amplitude attribute can provide information on the location or shape of strike-slip fractures, relying solely on this attribute has limitations in accurately identifying strike-slip faults. Therefore, this paper employs a multi-attribute fusion method, significantly improving the accuracy of strike-slip fracture identification.

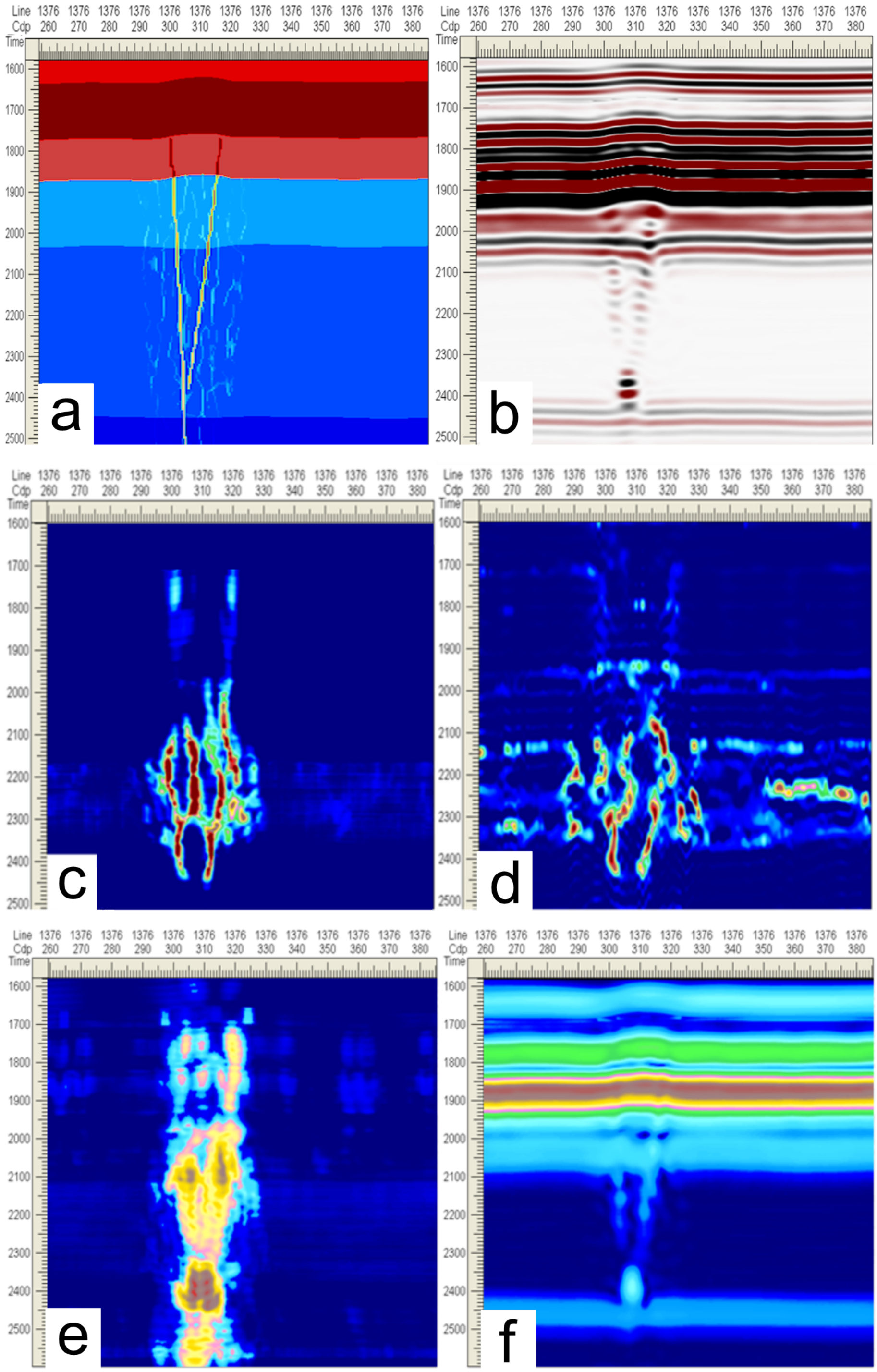

Figure 2. Attribute fracture recognition profile compared to the forward model profile

2.1.3. Attribute Fusion

Currently, commonly used methods within attribute fusion mainly include pattern recognition, ratio fusion, kernel principal component analysis, etc. Among these, ratio fusion adds weight to different attribute data according to certain proportions, obtaining final results. Comparatively speaking, ratio fusion operation is convenient and fast, while the effect is also relatively excellent. Based on previous analysis, this paper optimizes gradient structure tensor amplitude attributes for attribute fusion.

Since the value range difference between structure gradient tensor amplitude attributes is extremely large, direct fusion will cause the structure gradient tensor feature to be covered,

making it unable to accurately identify local structural features within strike-slip fractures from fused property data. Therefore, we must first normalize structure gradient tensor amplitude properties, scaling both property value ranges into the same interval. This ensures that during the data fusion process, complete feature retention is possible for strike-slip fractures.

Finally, after the normalization treatment of gradient structure tensor amplitude properties, the two earthquake property volumes are combined according to a 1:1 proportion relationship ratio, generating a new fused property volume relative to a single property volume. This volume is better able to accurately identify strike-slip fractures.

2.2. Fracture Probability Calculation Based on Fused Attributes

In fused property images, faults are usually identified through relatively low or high values. Therefore, picking faults from property images is viewed as a problem of finding the optimal path through global minimum or maximum values. Traditionally, earthquake property images can detect most fault locations, but they also highlight noise. Additionally, traditional earthquake property images exhibit poor fault feature continuity. Therefore, this paper innovatively uses a dynamic programming algorithm to efficiently extract the optimal path within a window range, calculating the fracture probability based on path extraction repetition rate property value. Eventually, this forms a strike-slip fracture probability image. While achieving high-precision identification for strike-slip fractures, it also improves continuity within the fracture image.

In order to extract continuous fault information from fault properties, this paper first transposes the image into a new space, where the vertical axis represents earthquake trace, and the horizontal axis represents depth. Additionally, it discretely selects a certain number of seed points within the fault property image to indicate the position where the fault surface passes. It sets zero value (minimum value) within a conical region above and below seed points and sets one value (maximum value) near seed points to better select the optimal fault passing through the global maximum fault property value.

Since transposing the image does not actually improve fault features within the image, before picking up fault surfaces, nonlinear smoothing technology is applied to smooth the property image. This involves obtaining a smoothed image using low slope constraint nonlinear smoothing, ensuring smooth continuity of earthquake property features while weakening noise, making fault features more obvious and continuous.

To solve the constrained maximization problem and find the optimal path, this paper uses a nonlinear smoothing forward integration backward tracking dynamic programming algorithm [19]. The first step in smoothing enhances features related to selected faults or paths while weakening noise features unrelated to faults. The smoothing filter is achieved by applying nonlinear integration to the input fault property image forward and backward.

The forward accumulation (from left to right) of the input image g[i,j] with slope constraint |j[i+1]-j[i]| can be implemented in the following way:

$$f[0,j] = g[0,j] \tag{1}$$

$$f[i,j] = g[i,j] + max[fi-d, j-1+k = i-d+1i-1gk, j-1; fi-1, j; fi-d, j+1+k = i-d+1i-1gk, j+1] \tag{2}$$

In the above formula, i=1,2,...,N-1; d is the integer closest to 1 / ; g[i,j] is an input fault attribute image; f[i,j] is the forward accumulated image. This forward accumulation can be regarded as a one-sided smoothing filter. Since the min function is used in the above equation, it is nonlinear. Similarly, the backward accumulation (from right to left) of the input image g[i,j] with slope constraint can be implemented as follows:

$$b[N-1,j] = g[N-1,j] \tag{3}$$

$$b[i,j] = g[i,j] + max[bi + d, j - 1 + k = i + 1i + d - 1gk, j - 1; fi + 1, j; fi + d, j + 1 + k = i + 1i + d - 1gk, j + 1] \quad (4)$$

In the above formula, d=[1 /], which is the integer closest to 1 / ; and b(i,j) is the backward accumulated image. Bilateral nonlinear smoothing is defined as a combination of forward and backward accumulation:

$$s[i,j] = fi, j + bi, j - g[i,j] \quad (5)$$

In this process, g[i,j] needs to be subtracted because it has been calculated in both f[i,j] and b[i,j]. By using forward and backward accumulation with slope constraints, low slope constraints can be imposed in nonlinear smoothing to produce smooth and continuous features in smoothed images. After smoothing, noise features are weakened, while image features related to faults are more obvious and continuous in the middle of the output image, making it easier for faults or maximum paths to be selected. In smoothing processing, the areas above and below the control points are set to zero values, while values near the control points are set to 1 to ensure that the next step of maximum path picking will find a path through the control points. Through the above processing, local optimal paths passing through each seed point can be obtained. Most of these selected paths correctly follow fault attribute features, where some paths traversed multiple times indicate that they may follow real faults. Paths traversed by a few or only once indicate that the path is a false image caused by noise rather than a real fault. The higher the overlap of paths, the higher the probability of fracture. In this paper, attribute values and path overlap are comprehensively integrated to calculate fracture probability using the following formula.

$$k^*[i,j] = ki, j - kminkmax - kmin \quad (6)$$

$$g^*[i,j] = \frac{g[i,j] - g_{min}}{g_{max} - g_{min}} \quad (7)$$

$$P = \begin{bmatrix} k^*_{11} & \cdots & k^*_{m1} \\ \vdots & \ddots & \vdots \\ k^*_{1n} & \cdots & k^*_{mn} \end{bmatrix} \begin{bmatrix} g^*_{11} & \cdots & g^*_{m1} \\ \vdots & \ddots & \vdots \\ g^*_{1n} & \cdots & g^*_{mn} \end{bmatrix} \quad (8)$$

In the above formula, k[i,j] represents the number of path traversals, g[i,j] represents the input attribute value, and P represents the calculated fracture probability.

3. Fracture Identification Based on Forward Modeling Data

In this paper, we employ seismic forward modeling and the multi-attribute optimal surface election fracture identification technology. This approach involves the fusion of selected amplitude attribute data with gradient structure tensor attributes. Building upon this integration, we utilize an optimal surface voting algorithm for strike-slip fracture identification.By comparing the results of strike-slip fracture identification with forward models (refer to Figure 3), it is evident that the optimal surface voting algorithm accurately characterizes fracture locations. Moreover, it effectively distinguishes between two closely positioned fractures. Simultaneously, this method proves capable of suppressing noise, providing a clearer and more continuous characterization of fracture distribution."Feel free to let me know if you have any specific preferences or if you would like further adjustments!

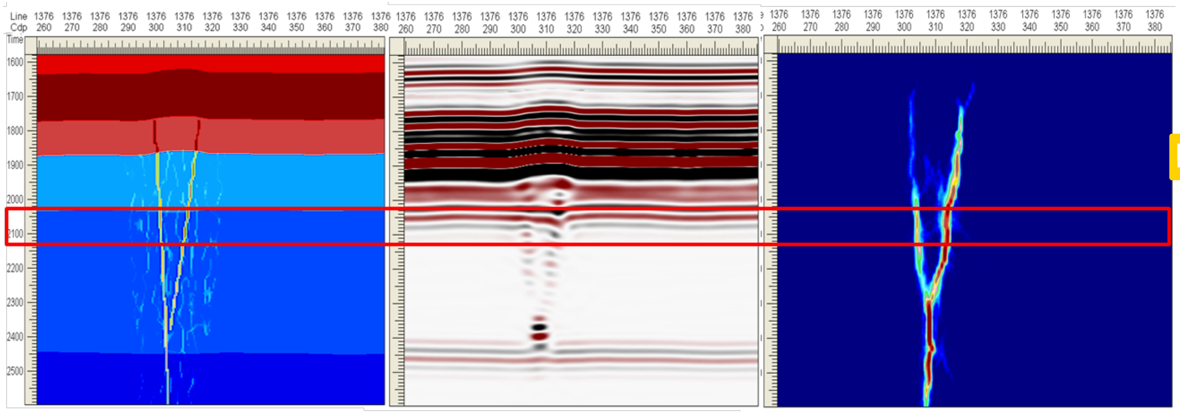

Figure 3. Comparison of optimal surface voting fracture identification profile with forward model profile (a)Forward model profile (b)Seismic record profile Figure(C)Optimal surface voting fracture identification profile .

On this basis, this paper performs thinning processing on seismic records to obtain a three-dimensional seismic data volume with a trace spacing of 25m, and on this basis uses optimal surface voting fracture identification technology for fracture detection. From Figure 4 it can be seen that as trace spacing increases, fracture detection results are significantly affected.

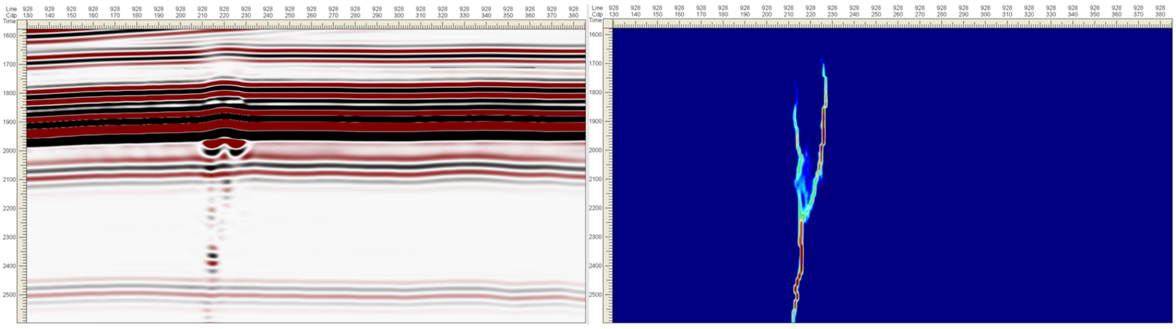

Figure 4. Comparison of seismic record with optimal surface voting fracture identification profile with 25m trace spacing (a)Seismic record profile with 25m trace spacing (b)Optimal surface voting fracture identification profile with 25m trace spacing .

The impact of trace spacing on fracture detection results is evident, with smaller trace spacing leading to higher recognition accuracy. When seismic records with a trace spacing of 25m were analyzed, the fracture detection identified two closely positioned fractures as a single fracture, and small fractures could not be accurately identified. However, under conditions of a trace spacing of 12.5m, the recognition accuracy significantly improved.This paper conducted fracture detection along the Ying Mountain Group (TO12y) and Peng Luan Group (TO1p) by extracting equal-depth slices from the optimal surface voting fracture identification data volume. These results were then compared with forward model stratum slices (refer to Figure 5), revealing a substantial alignment between fracture detection results and forward models."Feel free to let me know if you have any specific preferences or if you would like further adjustments!

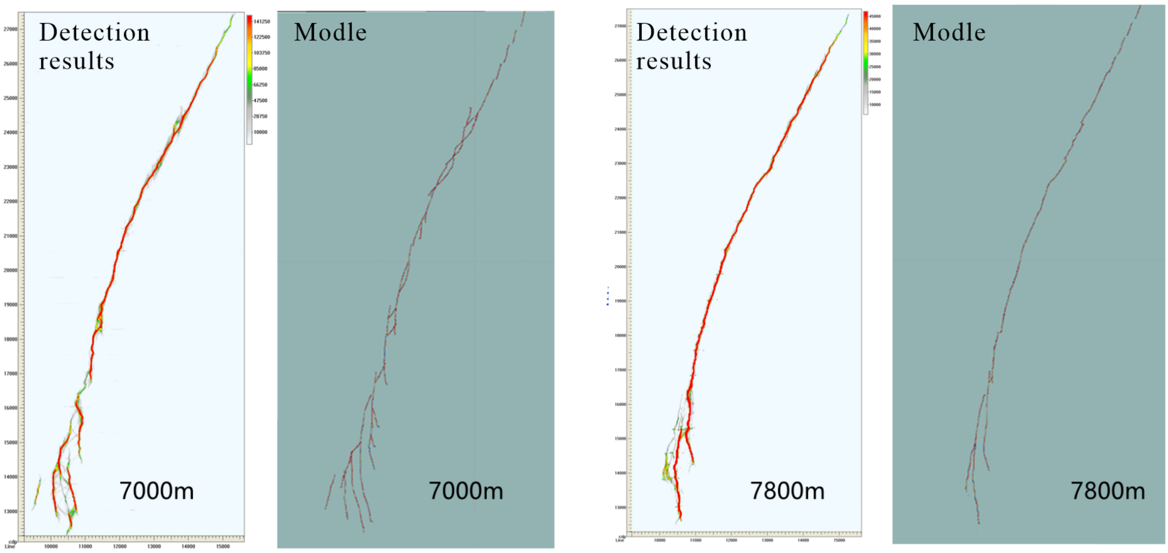

Figure 5. Comparison of equal depth slices from optimal surface voting fracture identification with forward model slices .

4. Real Data Processing

The Tarim Basin, a vast composite basin comprising Paleozoic craton basins and Mesozoic-Cenozoic foreland basins, stands out with its extensive Ordovician carbonate reservoirs, making it China's largest inland oil and gas basin. During its early exploration and development, the emphasis was primarily on ancient uplifts. However, with significant advancements in the understanding of deep-ultra deep Ordovician carbonate reservoirs, attention has shifted to fault-controlled dissolution body oil and gas reservoirs, marking a focal point in oilfield exploration and development [20-21]. The focus on reservoir understanding has gradually transitioned to fault-controlled storage and control.In this context, the identification of strike-slip faults assumes crucial significance for guiding oil and gas resource exploration and development. This paper takes the Yueshan area as an example to assess the application effectiveness of the multi-attribute optimal surface voting fault identification technology proposed herein.The model forward data profiles exhibit characteristics similar to actual seismic data and effectively capture diverse seismic anomaly characteristics of strike-slip fault zones within the study area (refer to Figure 6). Therefore, attribute selection based on model data serves as a valuable reference for the identification of strike-slip faults in actual data.

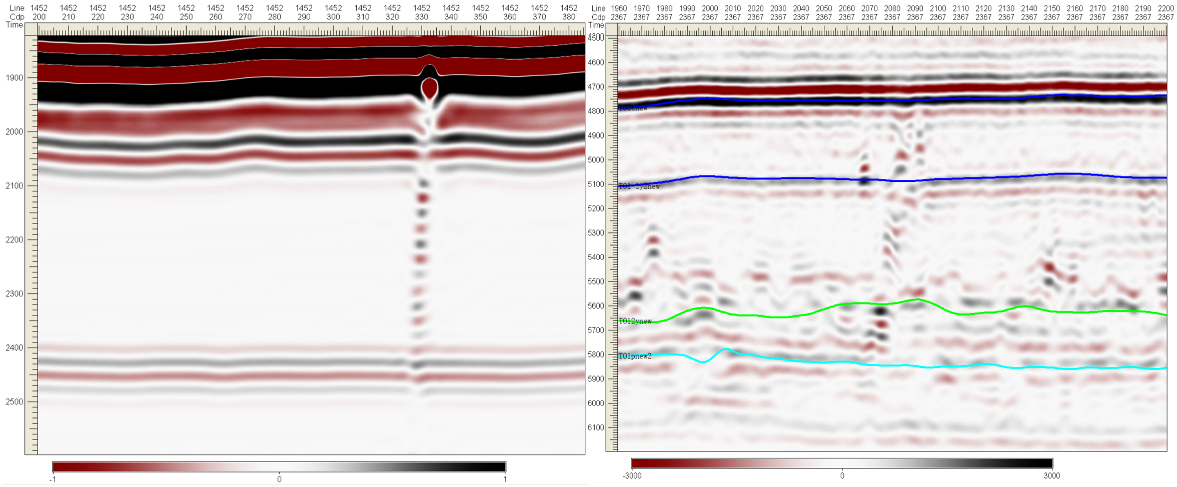

Figure 6. Comparison of forward simulated seismic data profile with actual seismic https://www.overleaf.com/project/646cefd96881a092405f1739data profile The target layer segment seismic data quality in study area is poor,;(a) Actual seismic data profile;(b) Forward simulated seismic data profile .

The target layer segment seismic data quality in study area is poor. Fault breakage zones show uneven energy and chaotic reflection characteristics on seismic profiles. Some strike-slip faults are not clearly identified and fault distance is not obvious. Low-speed zones caused by faults cause bead-like reflections (Figure 7).

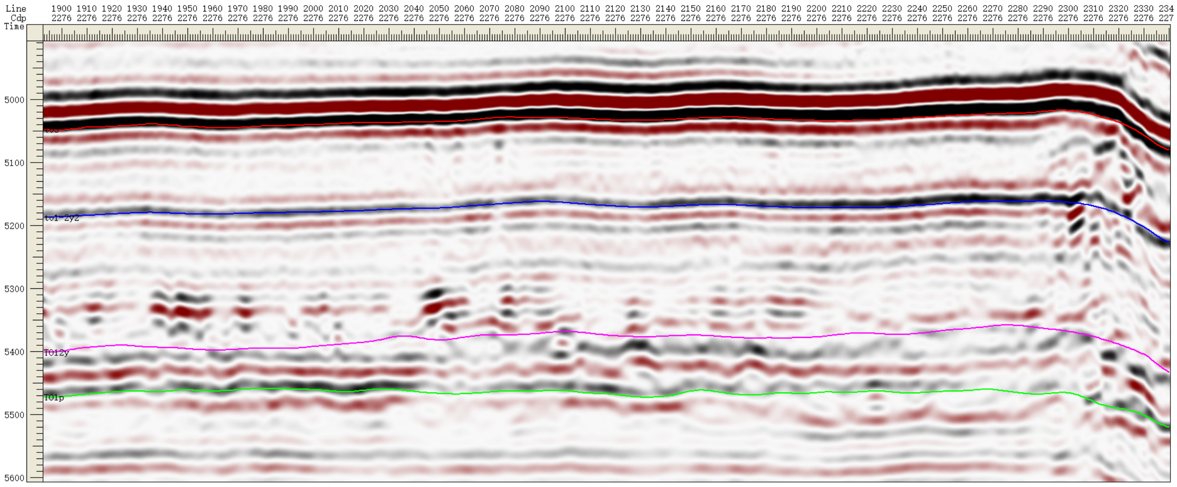

Figure 7. Tarim Basin area seismic record (profile) .

In addressing the diverse seismic anomalies in the Yueshan area's strike-slip fault zone, where a single attribute falls short in fully characterizing the complete features of the strike-slip fault, we integrated the test results from forward models. Specifically, amplitude attributes were selected, as they demonstrated superior identification of bead-like reflection amplitude anomalies. This selection facilitated the determination of fault location and morphology. Concurrently, high values from the gradient structure tensor were found to align with the reflection anomaly areas of dissolution bodies, indicating the comprehensive influence of various elements within the dissolution body configuration. To enhance strike-slip fault identification, we employed the optimal surface voting algorithm based on the fusion of attribute data volumes (refer to Figure 8).

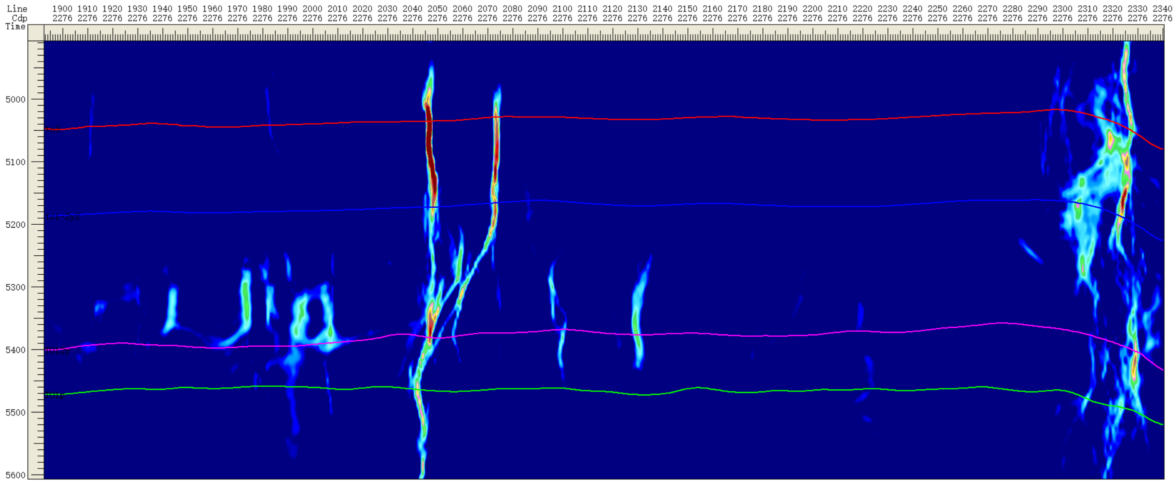

Figure 8. Tarim Basin area multi-attribute optimal surface voting fault identification data volume (profile) .

Overlaying Tarim Basin Yueman Region strike-slip fault identification data volume with seismic record data (Figure 9), it can be seen that this technology can effectively identify subtle waveform changes caused by strike-slip faults while suppressing noise achieving accurate characterization of strike-slip faults.

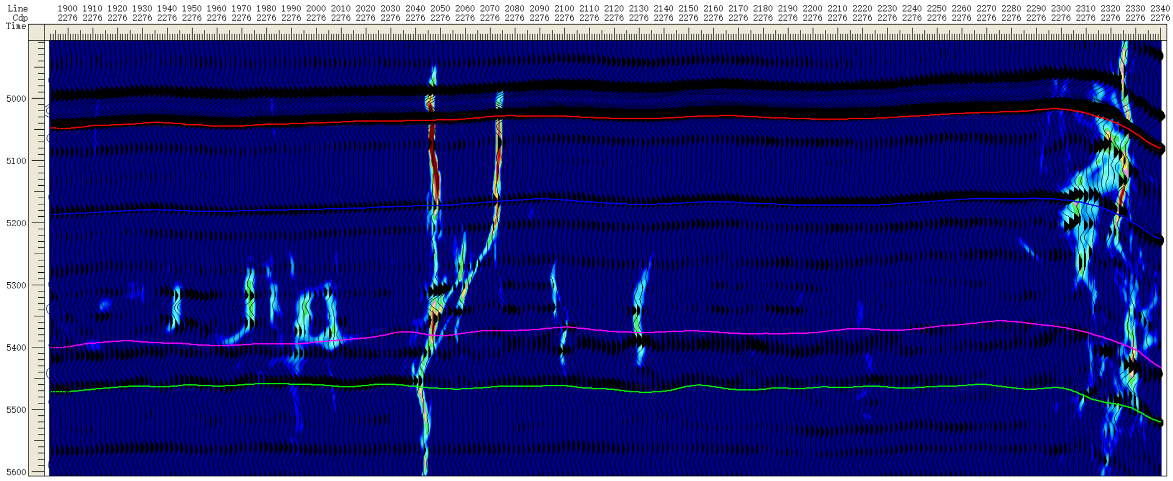

Figure 9. Tarim Basin area strike-slip fault identification data volume overlaid with seismic record (profile) .

Along Ying Mountain Group (TO12y) and PengLai Group (TO1p), multi-attribute optimal surface voting fault identification slices were extracted (Figure 10). Noise in original data was suppressed while continuity was strong in fault identification results with high accuracy.

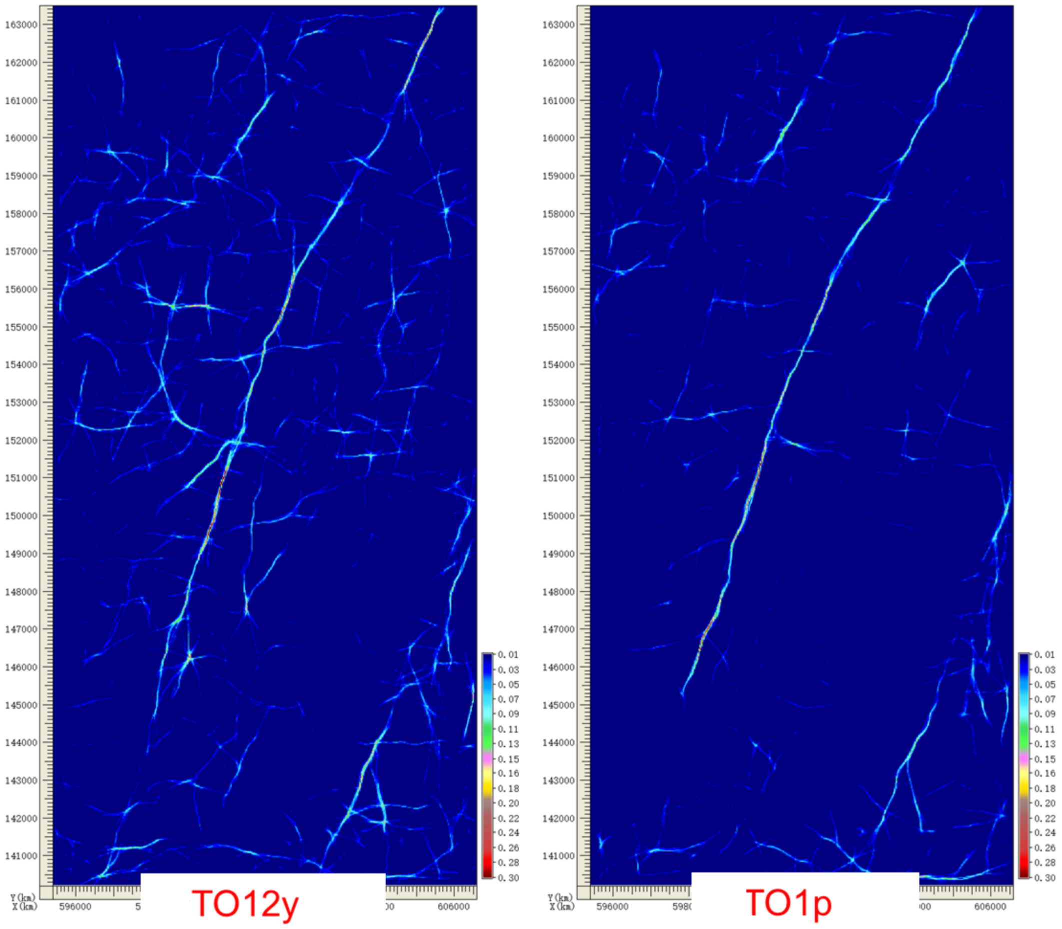

Figure 10. Tarim Basin Yueshan area strike-slip fault identification along layer slice .

Based on strike-slip fault identification results profile passing through well Y2 was extracted within study area (Figure 11). Combined with logging interpretation data and actual production data well Y2 encountered strike-slip fault zone obtaining oil layer thickness of 9.5m cumulative production exceeding 100000 tons. Therefore it can be seen that method combining fusion attributes with dynamic optimization optimal surface recognition algorithm can achieve accurate recognition of strike-slip faults providing technical guarantee for exploration and development in Yueman area.

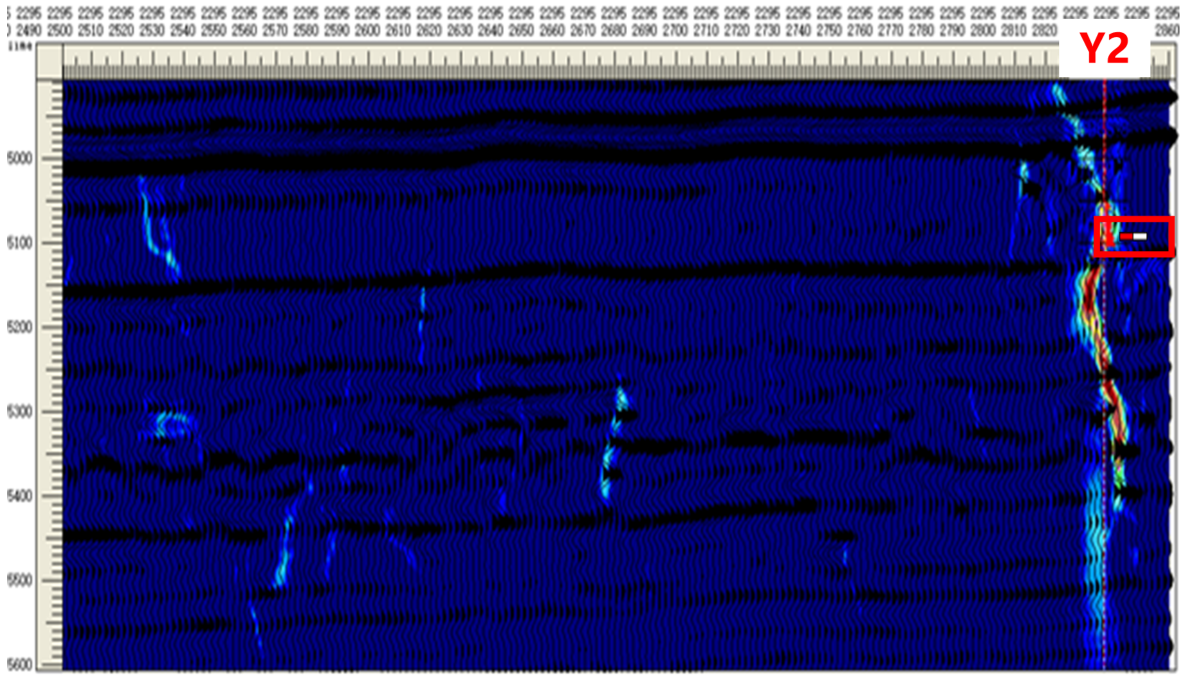

Figure 11. Strike-slip fault identification profile passing through well Y2 within study area .

5. Results

Strike-slip faults typically exhibit minimal observable changes in distance on seismic profiles, and their structures generally do not cause interference or amplitude alterations in reflected waves. This makes the identification of strike-slip faults challenging using traditional seismic processing methods such as coherence, variance, curvature, etc.

To address the diverse seismic anomalies within strike-slip fault zones, where a single attribute falls short in fully characterizing the complete features of strike-slip faults, this paper, based on forward simulated records, focused on selecting amplitude attributes. These attributes proved more effective in identifying bead-like reflection amplitude anomalies, aiding in the determination of location and morphology. Additionally, high values from the gradient structure tensor consistently aligned with reflection anomaly areas of dissolution bodies, reflecting the comprehensive influence of various elements within the dissolution body configuration.

In practical application within the Tarim Basin's Yueshan area exploration and development, particularly in controlled type carbonate rock reservoirs, the method successfully identified subtle waveform changes caused by strike-slip faults while suppressing noise. This approach achieved accurate characterization of strike-slip faults, providing a technical guarantee for the exploration and development of the Yueshan area.

References

V Baqués . Fracture, Dissolution, and Cementation Events in Ordovician Carbonate Reservoirs, Tarim Basin, NW China .*Geofluids* **2020**, *243*, 1-28.

Hale.D.. Seismic characterization of a carbonate reservoir in Tarim Basin. . *Geophysics* **2017**, *825*, B177-B188 .

Xingliang Deng . Characteristics and well location deployment ideas of strike-slip fault controlled carbonate oil and gas reservoirs: A case study of the Tarim Basin . *Natural Gas Industry* **2021**, *413*, 21-29.

Hale.D.. 3-Dseismic attributes using a semblance-based coherency algorithm . *Geophysics* **1998**, *63*, 1150-1165.

Hale.D.. Coherency calculations in the presence of structural dip . *Geophysics* **1999**, *64*, 104-111.

Li.F . Coherence attribute at different spectral scales. *Interpretation* **2014**, *21*,SA99-SA106.

Van Bemmel . Seismic signal processing method and apparatus for generating a cube of variance values. *U.S.Paten***2000**.

Roberts.A. . Curvature attributes and their application to 3D interpreted horizons. *First Break* **2001**, *19*, 85-100.

Hale.D.. Baqués, V. Fracture, 3D volumetric multispectral esti mates of reflector curvature and rotation. *Geophysics* **2006**, *71NO.5*, 41-51.

Aqrawi.A. A. . Improved fault segmentation using a dip guided and modified 3D Sobel filter: 81st Annual International Meeting . *SEG* **2011**, *Expanded Abstracts*, 999–1003 .

Xiangwen Li . Characteristic of the strike-slip faults system and effect of faults on reservoir and hydrocarbon accumulation in Tazhong area, China. *Geophysical Prospecting for Petroleum* **2018**, *575*, 764-774.

Xiaoqing Cui . The Seismic Discontinuity Feature Enhancement Method Based on Tensor Voting. *Oil Geophysical Prospecting* **2021**, *566*,1351-1358.

Hale.D.. Eigenstructure-based coherence computations as an aid to 3-D structural and stratigraphic mapping. *Geophysics* **1999**, *64*, 1468–1479.

Neff.D. B. . Image feature extraction . *U.S. Patent* **2011**.

Neff.D. B. . Automated seismic fault detection and picking. *U.S. Patent* **2000**.

Hale.D.. Liu, Y. Seismic characterization of a carbonate reservoir in Tarim Basin. In *Geophysics* **2006**, *NO.4*, 21–27.

Hale.D.. Methods to compute fault images, extract fault surfaces, and estimate fault throws from 3D seismic images.*Geophysics* **2013b**, *NO.2*, O33–O43.

Wu Xinming . Automatic fault interpretation using optimal surface voting.*Paper presented at the 2018 SEG International Exposition and Annual Meeting*

Hale.D.. Dynamic warping of seismic images.*Geophysics* **2013a**, *NO.2*, S105–S115.

Zhiwen Ding. Origin, hydrocarbon accumulation and oil-gas enrichment of fault-karst carbonate reservoirs: A case study of Ordovician carbonate reservoirs in South Tahe area of Halahatang oilfield, Tarim Basin.*Petroleum Exploration and Development* **2020**, *4702*, 286-296.

Xueqin Zhao. The effect of the reservoir space in carbonate formation by fracture:Take the Ordovician carbonate formation in Lungudong region of Tarim Basin as an example .*Journal of Geology Science* **2010**, *4501*, 292-306.
