# Supplementary material for: Application of a dynamic optimization-based multi-attribute fusion method for fault detection
Source: PLoS One. 2025 Mar 17;20(3):e0311079. doi: 10.1371/journal.pone.0311079 (PMC11913290; doi:10.1371/journal.pone.0311079)
Supplement: Attachment — Submitted filename: reviewers recommendation.docx [file pone.0311079.s002.docx]

**General comment**

I carefully read the paper by Chen Ma et al., in which the authors investigate the identification of strike-slip faults in ultra-deep dense limestone in the Yuemeng area of the Tarim Basin, China. They focus on improving the accuracy and continuity of fracture identification in order to enhance oilfield production and guide oil and gas resource exploration and development. The study proposes a multi-attribute optimal surface-based fracture identification technology, based on forward simulation records, to suppress noise and identify subtle waveform changes caused by strike-slip faults. The research aims to address the challenges of accurately identifying strike-slip faults in complex seismic reflection characteristics and chaotic seismic noise. Although the work is interesting, major adjustments are necessary to enhance the scientific merit of the paper to be published. It's clear that these revisions are crucial for the paper's appropriateness for publication in a prestigious and impactful journal such as PLOS ONE. To better align the manuscript with the journal's objective of increasing the pertinence of research, I recommend a series of modifications, including, to cite relevant and up-to-date bibliographical works (which are particularly deficient in introducing the research question and the gaps to be bridged with this research), the use and fluency of English language, improve the quality of images and especially the captions of these, draft a paragraph regarding the geographical/geological framework of the study area (that is totally lacking), Restructuring the paper according to the classical structure known as IMRaD (Introduction - Method - Results - and - Discussion). In the specific comments and in the PDF file with annotations and suggestions the authors will find additional advices.

**Specific comment**

The scientific research article is highly intriguing, also perhaps because of my scientific background; however, some major modifications are needed to refine the paper presentation and clarity of the concepts discussed. Authors will find the following comments also in the attached pdf file to be facilitated in revisions.

1-I recommend the authors to modify the title of the paper in “Application of a Dynamic Optimization-Based Multi-Attribute Fusion Method for Fault Detection”, which is clearer and more concise.

2-In the abstract is mentioned the Yueman area. Since the Tarim Basin covers an area of 888,000 km2, and since there is no geographic (with an attached location map) and geological framework in the paper, it would be explanatory to at least place indications of the cardinal points of this area.

3-row 6: rephrase “[…] strike-slip fault zones in ultra-deep dense limestone is a pressing issue […]” in “strike-slip fault zones in ultra-deep tight limestone is a critical issue”.

4-row 8: I do not understand what the authors mean by 'beaded'. I suggest finding a clearer synonym.

5-row 12: perhaps the term 'merged' is more appropriate than 'fused'.

6-rows 16-18: this sentence is not useful as the location has already been declared as well as the visualisation is improved. I advise the authors to delete it.

7-About the Keywords: I advise the authors to work better on keywords. Since search engines often identify works by their title, abstract and keywords, authors will be more likely to be read if they use the right and non-repeated keywords among the search items. E.g., "strike-slip fault" is already present three times in the abstract, maybe another keyword could be more suitable.

8- At the very beginning of the Introduction paragraph, where authors state “[…] The deep-seated strike-slip faults in western China play a pivotal role in controlling substantial reserves of oil and gas. Both the main fault zone and secondary faults along the strike-slip fault exhibit enhanced displays of oil and gas, demonstrating robust fault-controlled storage and enrichment characteristics. […]”, Citations are needed to support theese sentences. Recommendation:

- Zhao, R., Zhao, T., Kong, Q., Deng, S. and Li, H., 2020. Relationship between fractures, stress, strike-slip fault and reservoir productivity, China Shunbei oil field, Tarim Basin. Carbonates and Evaporites, 35, pp.1-14.

- Deng, S., Zhao, R., Kong, Q., Li, Y. and Li, B., 2022. Two distinct strike-slip fault networks in the Shunbei area and its surroundings, Tarim Basin: Hydrocarbon accumulation, distribution, and controlling factors. AAPG Bulletin, 106(1), pp.77-102.

9- Continuing in the introductions, where authors state “[…] Nevertheless, exploring ultra-deep fault-controlled carbonate reservoirs proves to be exceedingly complex, particularly within the strike-slip fault fracture zone where reservoirs exhibit significant heterogeneity. Reservoir characteristics suggest that storage spaces are predominantly influenced by dissolution or tectonic factors. […]”, Also this sentence needs citations, authors should cite:

- Tomassi, A., Trippetta, F., de Franco, R. and Ruggieri, R., 2023. How petrophysical properties influence the seismic signature of carbonate fault damage zone: Insights from forward-seismic modelling. Journal of Structural Geology, 167, p.104802.

- Tomassi, A., Milli, S. and Tentori, D., 2024. Synthetic seismic forward modeling of a high-frequency depositional sequence: The example of the Tiber depositional sequence (Central Italy). Marine and Petroleum Geology, 160, p.106624.

10- in rows 27-29, when authors state “[…] Reservoir characteristics suggest that storage spaces are predominantly influenced by dissolution or tectonic factors. […]”, usually, in scientific publications, introductions and discussions are the most citation-rich paragraphs. Especially in introductions in which the research question is contextualised in the international scenario, almost every sentence written by the authors has to be justified by the literature unless it is a 'discovery' of the authors themselves illustrated for the first time in the paper in question. It seems strange to me how there is this enormous lack of citations here. I give a tip on citation:

- Radwan, A.E., Trippetta, F., Kassem, A.A. and Kania, M., 2021. Multi-scale characterization of unconventional tight carbonate reservoir: Insights from October oil filed, Gulf of Suez rift basin, Egypt. Journal of Petroleum Science and Engineering, 197, p.107968.

11- row 37: I do not understand what the authors mean by energy 'beads'. Perhaps that these reflectors have marked amplitudes caused by the strong acoustic impedance contrast?

12- in rows 37-38, The authors should explain why these reflectors denote high-quality reservoir (even referring to literature).

13- in row 39 authors state “[…] existing technologies excel in accurately identifying strong ’beads’ reflections […]”, in my opinion authors should mention which technologies.

14- The second paragraph should be a geological setting section. The authors need to add a paragraph on the geographical-geological-stratigraphic-geodynamic framework of the area under examination, which is totally lacking. A case study such as the one examined in this paper certainly deserves to be valorised in order to also contextualise the research and be applicable (and reproducible) to other similar realms worldwide, also giving a broader scope to the work.

15- in rows 76-77, authors state “[…] Forward simulations are then conducted to serve as a foundation for attribute optimization in strike-slip fault identification. […]”. When stating concepts already well established in the literature, authors are requested to add references. Authors should cite here:

- Alaei, B., 2012. Seismic modeling of complex geological structures. Seismic Waves-Research and Analysis, 11, pp.528-529.

- Tomassi, A., Trippetta, F., de Franco, R. and Ruggieri, R., 2022. From petrophysical properties to forward-seismic modeling of facies heterogeneity in the carbonate realm (Majella Massif, central Italy). Journal of Petroleum Science and Engineering, 211, p.110242.

- De Franco, R., Petracchini, L., Scrocca, D., Caielli, G., Montegrossi, G., Santilano, A. and Manzella, A., 2019. Synthetic seismic reflection modelling in a supercritical geothermal system: An image of the k-horizon in the larderello field (Italy). Geofluids, 2019.

16- in paragraph “2.1.1. Establishment of Forward Model”, It would be very interesting if the authors also stated the central frequency of acquisition and the vertical and horizontal resolution, which are decisive factors in fault detection especially when dealing with very deep sub-vertical faults. Authors are requested to add this information.

17- Figure1: This caption is very unexplanatory. The authors should also briefly describe what is seen in the volumes represented. They should also state in the image which is Figure 1A and which is Figure 1B.

18- in rows 117 to 119, authors state: “[…] The paper investigates the seismic response characteristics in different reservoir configurations based on typical fracture-controlled carbonate rock models and forward data. […]”. I understood this to be the case study of the Tarim Basin. Perhaps the authors meant that this workflow is also applicable to other similar situations in other contexts. The authors need to clarify this statement.

19- The sentence from row 121 to row 123 is redundant and repetitive of what the authors have already written many times before.

20- from row 124 to row 169, where the authors list the attributes, the symbol ':' is missing for each attribute between its definition and description.

21- from row 151 to 153, where the authors state “[…] Seismic faults usually cause phenomena such as reflection or refraction when waves propagate underground thus forming different amplitude sizes in waveforms. […]”, What about diffractions?

22- Authors should first describe Figure2A, then Figure 2B, then 3C, and so on in order to avoid reader confusion. Furthermore, I wonder why Figure 2B is not described.

23- in row 170, I do not understand what the authors mean by 'bead-like' reflection.

24- Caption of Figure 2 is very unexplanatory. The authors should describe what is seen in the different insets of the image represented. They should also state in the image which is Figure 2A, which is Figure 2B, 2C... and so on.

25- About the sentences from row 196 to row 203: I don't know if I understood correctly: thus, can the methods in this paper predict where a new seismogenic fault will occur? If yes, I hope the authors will describe this later in the paper as it has very important implications.

26- rows 249-251: The authors have already stated this several times in the narrative of the text. Consequently, they are requested to be less repetitive.

27- the sentence at rows 257-258 “[…]"Feel free to let me know if you have any specific preferences or if you would like further adjustments! […]” I think it is a copy-paste error from some other document.

28- The image reported in Figure 3 should report which is Figure 3A, which 3B, which 3C.

29- The image reported in Figure 4 should report which is Figure 4A and which 4B.

30- in rows 268-270 when authors state “[…] This paper conducted fracture detection along the Ying Mountain Group (TO12y) and Peng Luan Group (TO1p) […]”, these things are never introduced to the reader, so it is not clear what the authors are talking about. I suggest again that the authors should draft an accurate geological and geographical background to be added after the introductory paragraph with an attached location map.

31- Again, the sentence at rows 273-274 “[…]"Feel free to let me know if you have any specific preferences or if you would like further adjustments! […]” I think it is a copy-paste error from some other document.

32- Again, the caption of Figure 5 is very unexplanatory. The authors should describe what is seen in the different insets of the image represented. They should also state in the image (and the caption as well) which is Figure 5A, which is Figure 5B, 5C, and 5D.

33- The sentence in rows 276-278 is one of those sentences that I think should go in the geological framework.

34- row 285 “[…] This paper takes the Yueshan area […]”. But wasn't it Yueman area? this is unclear, I repeat that the paper definitely needs a geological framework to make the work much clearer. Also in row 289 is stated “[…] within the study area […]” but study area is unclear.

35- Again, the sentence in rows 290-291 is repetitive and redundant.

36- in Figure 6, authors must declare which is Figure 6A and which 6B in the image.

37- Caption of figure 6: Clicking on the link proposed in the caption (which I assume is the source of the seismic line even though it is not stated by the authors that it should be, the reader must deduce many things for himself) opens an unreachable page (Not found. Sorry, we can't find the page you are looking for) ...

By fixing the link (as the word 'data' is not separated from the url) the page is still unreachable (Restricted, sorry you don't have permission to load this page.). I therefore wonder what sense there is in putting the link in the caption.

38- row 294. Perhaps the authors mean low-velocity instead of “low-speed”.

39- row 295. I do not understand what the authors mean by 'bead-like'.

40- Authors must implement the caption of Figure 7 by describing what they want to show in the figure in detail. A caption presented in this way is not explanatory.

41- row 310: “[…] Along Ying Mountain Group (TO12y) and PengLai Group (TO1p) […]”. I repeat that the study area is unclear.

42- Authors are requested to improve the caption and the Figure 10 by following the suggestions provided for the previous images.

43- row 314: “[…] Based on strike-slip fault identification results profile passing through well Y2 […]”. A base map of the wells is needed as they are not even marked in the profiles.

44- Section “5.Results”. This is actually the conclusion paragraph (both in terms of location and the concepts written in it). The results paragraph is the one shown above. I advise the authors to follow the so-called IMRaD format for the structure of the paper. IMRaD is an acronym for Introduction - Method - Results - and - Discussion.
