# Supplementary material for: Application of a dynamic optimization-based multi-attribute fusion method for fault detection
Source: PLoS One. 2025 Mar 17;20(3):e0311079. doi: 10.1371/journal.pone.0311079 (PMC11913290; doi:10.1371/journal.pone.0311079)
Supplement: Attachment — Submitted filename: Response to Reviewer.docx [file pone.0311079.s003.docx]

**First, I want to explain the files I submitted: The submitted files consist of an annotated original file and the revised manuscript. The two are not exactly the same. The annotated original file retains the annotations for modifications based on the original PDF without changing the format or expression. Only the issues mentioned in the comments were addressed, and modifications and responses were made accordingly. However, as per the requirements of the editorial department, the manuscript needs to be polished by a professional scientific editor to improve language expression and modify the format to achieve the best presentation. Therefore, the manuscript is the result of polishing in terms of format and expression.**

**Below, I will respond to the questions raised by the two reviewers and provide information on the modifications made.**

**Reviewer 1**

1-I recommend the authors to modify the title of the paper in “Application of a Dynamic Optimization-Based Multi-Attribute Fusion Method for Fault Detection”, which is clearer and more concise.

*Following the advice of the peer reviewer, I have completed the revisions to the title.*

2-In the abstract is mentioned the Yueman area. Since the Tarim Basin covers an area of 888,000 km2, and since there is no geographic (with an attached location map) and geological framework in the paper, it would be explanatory to at least place indications of the cardinal points of this area.

*I have added a paragraph describing the geological framework of the study area in the manuscript and marked the approximate location of the study area. However, due to confidentiality reasons, I cannot provide precise geographical location information. I apologize for any inconvenience.*

3-row 6: rephrase “[…] strike-slip fault zones in ultra-deep dense limestone is a pressing issue […]” in “strike-slip fault zones in ultra-deep tight limestone is a critical issue”.

*Thank you for your correction. I have rewritten it as per your request.*

4-row 8: I do not understand what the authors mean by 'beaded'. I suggest finding a clearer synonym.

"beaded" refers to a specific reflection pattern observed on seismic profiles, characterized by a series of high-energy points or areas, resembling a beaded pattern.

*"beaded" refers to a specific reflection pattern observed on seismic profiles, characterized by a series of high-energy points or areas, resembling a beaded pattern.*

5-row 12: perhaps the term 'merged' is more appropriate than 'fused'.

*Thank you for your suggestion.*

6-rows 16-18: this sentence is not useful as the location has already been declared as well as the visualisation is improved. I advise the authors to delete it.

*Thanks for your reminder. I have deleted this paragraph.*

7-About the Keywords: I advise the authors to work better on keywords. Since search engines often identify works by their title, abstract and keywords, authors will be more likely to be read if they use the right and non-repeated keywords among the search items. E.g., "strike-slip fault" is already present three times in the abstract, maybe another keyword could be more suitable.

*Thanks for the recommendation of the reviewer, I have read the article you recommended, which can indeed provide more perfect data support, so I add new references.*

8- At the very beginning of the Introduction paragraph, where authors state “[…] The deep-seated strike-slip faults in western China play a pivotal role in controlling substantial reserves of oil and gas. Both the main fault zone and secondary faults along the strike-slip fault exhibit enhanced displays of oil and gas, demonstrating robust fault-controlled storage and enrichment characteristics. […]”, Citations are needed to support theese sentences. Recommendation:

- Zhao, R., Zhao, T., Kong, Q., Deng, S. and Li, H., 2020. Relationship between fractures, stress, strike-slip fault and reservoir productivity, China Shunbei oil field, Tarim Basin. Carbonates and Evaporites, 35, pp.1-14.

- Deng, S., Zhao, R., Kong, Q., Li, Y. and Li, B., 2022. Two distinct strike-slip fault networks in the Shunbei area and its surroundings, Tarim Basin: Hydrocarbon accumulation, distribution, and controlling factors. AAPG Bulletin, 106(1), pp.77-102.

*Thanks for the recommendation of the reviewer, I have read the article you recommended, which can indeed provide more perfect data support, so I add new references.*

9- Continuing in the introductions, where authors state “[…] Nevertheless, exploring ultra-deep fault-controlled carbonate reservoirs proves to be exceedingly complex, particularly within the strike-slip fault fracture zone where reservoirs exhibit significant heterogeneity. Reservoir characteristics suggest that storage spaces are predominantly influenced by dissolution or tectonic factors. […]”, Also this sentence needs citations, authors should cite:

- Tomassi, A., Trippetta, F., de Franco, R. and Ruggieri, R., 2023. How petrophysical properties influence the seismic signature of carbonate fault damage zone: Insights from forward-seismic modelling. Journal of Structural Geology, 167, p.104802.

- Tomassi, A., Milli, S. and Tentori, D., 2024. Synthetic seismic forward modeling of a high-frequency depositional sequence: The example of the Tiber depositional sequence (Central Italy). Marine and Petroleum Geology, 160, p.106624.

*Thanks for the recommendation of the reviewer, I have read the article you recommended, which can indeed provide more perfect data support, so I add new references.*

10- in rows 27-29, when authors state “[…] Reservoir characteristics suggest that storage spaces are predominantly influenced by dissolution or tectonic factors. […]”, usually, in scientific publications, introductions and discussions are the most citation-rich paragraphs. Especially in introductions in which the research question is contextualised in the international scenario, almost every sentence written by the authors has to be justified by the literature unless it is a 'discovery' of the authors themselves illustrated for the first time in the paper in question. It seems strange to me how there is this enormous lack of citations here. I give a tip on citation:

- Radwan, A.E., Trippetta, F., Kassem, A.A. and Kania, M., 2021. Multi-scale characterization of unconventional tight carbonate reservoir: Insights from October oil filed, Gulf of Suez rift basin, Egypt. Journal of Petroleum Science and Engineering, 197, p.107968.

*Thanks for the recommendation of the reviewer, I have read the article you recommended, which can indeed provide more perfect data support, so I add new references.*

11- row 37: I do not understand what the authors mean by energy 'beads'. Perhaps that these reflectors have marked amplitudes caused by the strong acoustic impedance contrast?

*In "Energy 'beads'," "beads" refers to a specific reflection pattern observed on seismic profiles, typically characterized by a series of high-energy points or areas, resembling a beaded or string-like formation. In seismology, this reflection pattern is often associated with specific geological structures or lithological variations in underground rock formations.*

12- in rows 37-38, The authors should explain why these reflectors denote high-quality reservoir (even referring to literature).

*Thank you for your correction. I have added new references to improve the rigor of the article.*

窗体顶端

13- in row 39 authors state “[…] existing technologies excel in accurately identifying strong ’beads’ reflections […]”, in my opinion authors should mention which technologies.

*Yes, following your correction, I listed relevant methods at that location.*

14- The second paragraph should be a geological setting section. The authors need to add a paragraph on the geographical-geological-stratigraphic-geodynamic framework of the area under examination, which is totally lacking. A case study such as the one examined in this paper certainly deserves to be valorised in order to also contextualise the research and be applicable (and reproducible) to other similar realms worldwide, also giving a broader scope to the work.

*Thank you for pointing out the issue. I have added a section describing the geological background of the study area to enhance the completeness of the paper.*

15- in rows 76-77, authors state “[…] Forward simulations are then conducted to serve as a foundation for attribute optimization in strike-slip fault identification. […]”. When stating concepts already well established in the literature, authors are requested to add references. Authors should cite here:

- Alaei, B., 2012. Seismic modeling of complex geological structures. Seismic Waves-Research and Analysis, 11, pp.528-529.

- Tomassi, A., Trippetta, F., de Franco, R. and Ruggieri, R., 2022. From petrophysical properties to forward-seismic modeling of facies heterogeneity in the carbonate realm (Majella Massif, central Italy). Journal of Petroleum Science and Engineering, 211, p.110242.

- De Franco, R., Petracchini, L., Scrocca, D., Caielli, G., Montegrossi, G., Santilano, A. and Manzella, A., 2019. Synthetic seismic reflection modelling in a supercritical geothermal system: An image of the k-horizon in the larderello field (Italy). Geofluids, 2019.

*Thanks for the recommendation of the reviewer, I have read the article you recommended, which can indeed provide more perfect data support, so I add new references.*

16- in paragraph “2.1.1. Establishment of Forward Model”, It would be very interesting if the authors also stated the central frequency of acquisition and the vertical and horizontal resolution, which are decisive factors in fault detection especially when dealing with very deep sub-vertical faults. Authors are requested to add this information.

*Here, I have supplemented the center frequency and sampling rate used in the forward simulation.*

17- Figure1: This caption is very unexplanatory. The authors should also briefly describe what is seen in the volumes represented. They should also state in the image which is Figure 1A and which is Figure 1B.

*Thank you for your suggestion. I have made the modifications.*

18- in rows 117 to 119, authors state: “[…] The paper investigates the seismic response characteristics in different reservoir configurations based on typical fracture-controlled carbonate rock models and forward data. […]”. I understood this to be the case study of the Tarim Basin. Perhaps the authors meant that this workflow is also applicable to other similar situations in other contexts. The authors need to clarify this statement.

*Thank you for your suggestion. I have made the modifications.” The paper investigates the seismic response characteristics in different reservoir configurations based on typical fracture-controlled carbonate rock models and forward data. Various seismic attributes and characterization techniques are applied, and the results are compared and optimized to obtain the most suitable seismic attributes.*

*”*

19- The sentence from row 121 to row 123 is redundant and repetitive of what the authors have already written many times before.

*Thank you for your reminder. I have deleted this paragraph.*

20- from row 124 to row 169, where the authors list the attributes, the symbol ':' is missing for each attribute between its definition and description.

*I have added the symbol, thank you for your reminder.*

21- from row 151 to 153, where the authors state “[…] Seismic faults usually cause phenomena such as reflection or refraction when waves propagate underground thus forming different amplitude sizes in waveforms. […]”, What about diffractions?

*The expression here is indeed not rigorous enough. It has been supplemented, thank you for your correction.*

22- Authors should first describe Figure2A, then Figure 2B, then 3C, and so on in order to avoid reader confusion. Furthermore, I wonder why Figure 2B is not described.

*Here, I actually missed the descriptions for figures a and b. The description for figure a is only used to compare with figures c to f because figure a is the model image, making the comparison more direct. Thank you for pointing that out. I have now supplemented the descriptions for figures a and b.*

23- in row 170, I do not understand what the authors mean by 'bead-like' reflection.

"bead-like" refers to a pattern observed in seismic data where seismic reflections exhibit a series of localized high-amplitude peaks resembling beads on a string. These peaks typically indicate areas of strong reflectivity or impedance contrast in the subsurface.

24- Caption of Figure 2 is very unexplanatory. The authors should describe what is seen in the different insets of the image represented. They should also state in the image which is Figure 2A, which is Figure 2B, 2C... and so on.

*Thank you for your correction. I have made the necessary adjustments by adding descriptions for the images.*

25- About the sentences from row 196 to row 203: I don't know if I understood correctly: thus, can the methods in this paper predict where a new seismogenic fault will occur? If yes, I hope the authors will describe this later in the paper as it has very important implications.

*I'm sorry, but this paper does not predict where a new seismogenic fault will occur.*

26- rows 249-251: The authors have already stated this several times in the narrative of the text. Consequently, they are requested to be less repetitive.

*Thank you for your suggestion. I have already made the modifications according to your advice.*

27- the sentence at rows 257-258 “[…]"Feel free to let me know if you have any specific preferences or if you would like further adjustments! […]” I think it is a copy-paste error from some other document.

*Sorry, there were indeed some errors in the translation process.*

28- The image reported in Figure 3 should report which is Figure 3A, which 3B, which 3C.

*Here, I have made the changes according to your suggestion and added annotations.*

29- The image reported in Figure 4 should report which is Figure 4A and which 4B.

*Here, I have made the changes according to your suggestion and added annotations.*

30- in rows 268-270 when authors state “[…] This paper conducted fracture detection along the Ying Mountain Group (TO12y) and Peng Luan Group (TO1p) […]”, these things are never introduced to the reader, so it is not clear what the authors are talking about. I suggest again that the authors should draft an accurate geological and geographical background to be added after the introductory paragraph with an attached location map.

*Thank you for your suggestion. I will add geological and geographical background at the beginning of the actual data processing section.*

31- Again, the sentence at rows 273-274 “[…]"Feel free to let me know if you have any specific preferences or if you would like further adjustments! […]” I think it is a copy-paste error from some other document.

*Sorry, there were indeed some errors in the translation process.*

32- Again, the caption of Figure 5 is very unexplanatory. The authors should describe what is seen in the different insets of the image represented. They should also state in the image (and the caption as well) which is Figure 5A, which is Figure 5B, 5C, and 5D.

*Here, I have made the changes according to your suggestion and added annotations.*

33- The sentence in rows 276-278 is one of those sentences that I think should go in the geological framework.

*Thank you for your suggestion. I will add geological and geographical background at the beginning of the actual data processing section.*

34- row 285 “[…] This paper takes the Yueshan area […]”. But wasn't it Yueman area? this is unclear, I repeat that the paper definitely needs a geological framework to make the work much clearer. Also in row 289 is stated “[…] within the study area […]” but study area is unclear.

Sorry, I didn't notice the error at first; it was a typo.

35- Again, the sentence in rows 290-291 is repetitive and redundant.

*Thank you for your reminder. I have deleted this paragraph.*

36- in Figure 6, authors must declare which is Figure 6A and which 6B in the image.

*Here, I have made the changes according to your suggestion and added annotations.*

37- Caption of figure 6: Clicking on the link proposed in the caption (which I assume is the source of the seismic line even though it is not stated by the authors that it should be, the reader must deduce many things for himself) opens an unreachable page (Not found. Sorry, we can't find the page you are looking for) ...

By fixing the link (as the word 'data' is not separated from the url) the page is still unreachable (Restricted, sorry you don't have permission to load this page.). I therefore wonder what sense there is in putting the link in the caption.

*This is a typesetting system link, which allows for more efficient typesetting in a programmatic way. However, I inadvertently inserted the link into the document during export without realizing it. I apologize once again.*

38- row 294. Perhaps the authors mean low-velocity instead of “low-speed”.

*Thank you for your correction.*

39- row 295. I do not understand what the authors mean by 'bead-like'.

*"bead-like" refers to a pattern observed in seismic data where seismic reflections exhibit a series of localized high-amplitude peaks resembling beads on a string. These peaks typically indicate areas of strong reflectivity or impedance contrast in the subsurface.*

40- Authors must implement the caption of Figure 7 by describing what they want to show in the figure in detail. A caption presented in this way is not explanatory.

*Thank you for your feedback. We will revise the caption of Figure 7 to provide a more detailed description of the content depicted in the figure, ensuring that it is explanatory and informative.*

41- row 310: “[…] Along Ying Mountain Group (TO12y) and PengLai Group (TO1p) […]”. I repeat that the study area is unclear.

*The study area has been re-described in the manuscript to ensure that readers have a clearer understanding of the geographical and geological background of the research.*

42- Authors are requested to improve the caption and the Figure 10 by following the suggestions provided for the previous images.

*In Figure 10, annotations have been added, and the caption has been improved.*

43- row 314: “[…] Based on strike-slip fault identification results profile passing through well Y2 […]”. A base map of the wells is needed as they are not even marked in the profiles.

*In Figure 11, annotations have been added*

44- Section “5.Results”. This is actually the conclusion paragraph (both in terms of location and the concepts written in it). The results paragraph is the one shown above. I advise the authors to follow the so-called IMRaD format for the structure of the paper. IMRaD is an acronym for Introduction - Method - Results - and - Discussion.

*Thank you for your suggestion, indeed, this is the conclusion paragraph.*

**Reviewer 2**

1. Lack of acquisition parameters for the forward simulation model, which are crucial for readers to understand the effectiveness and reproducibility of the method.

*The paper has supplemented information on the sampling rate, dominant frequency of the wavelet, and other details.*

2. The parameter values provided during numerical experiments need to be explained in the text regarding the principles of selection, enabling readers to understand the rationale behind parameter choices.

*Supplementary explanations have been provided in the text. The selection of parameters is based on the collected data from the actual work area. Statistical wavelets are extracted from the target layer of the original seismic data, making the results of forward simulation seismic signals more closely approximate real conditions.*

3. The conventional attributes mentioned in the article need to be concisely introduced, specifying which attributes were specifically selected in this study and providing the rationale.

*In the text, explanations and comparisons of multiple attributes have been provided.*

4. Some figures lack legend explanations and need to be completed to enhance readers' understanding of the visual content.

*The missing legends, axes labels, and blurry images in the figures have been corrected, and the blurry images have been replaced.*

5. Non-original formulas in the text could be simplified to improve the fluidity and readability of the article.

*Thank you for your suggestion. We have simplified the formulas to the maximum extent possible.*

6. The conclusion section appears somewhat thin and could be enriched further, perhaps by discussing the limitations of the method or suggesting future research directions.

*Thank you for your suggestion.We have made adjustments to the conclusions to make them more substantial.*
