# Supplementary material for: Application of a dynamic optimization-based multi-attribute fusion method for fault detection
Source: PLoS One. 2025 Mar 17;20(3):e0311079. doi: 10.1371/journal.pone.0311079 (PMC11913290; doi:10.1371/journal.pone.0311079)
Supplement: Attachment — Submitted filename: Response to Reviewers.docx [file pone.0311079.s004.docx]

Dear Reviewer and Editor,

I hope this message finds you well.

I am writing to express my sincere gratitude for the invaluable feedback and suggestions provided by you and the reviewers regarding my manuscript. I have thoroughly addressed the comments and have made the necessary revisions to the conclusion section as requested. The revised manuscript and a marked-up version highlighting the changes have been uploaded for your review.

The professional insights offered by the reviewers have been immensely enlightening, significantly improving the quality of my research. I have gained a deeper understanding and appreciation for academic writing through this process. The detailed feedback has not only refined my manuscript but also enhanced my knowledge of the subject matter.

In addition to expanding the conclusion, I have also clarified the data analysis and methodology sections to ensure a more comprehensive and transparent presentation of the research process. I am confident that these revisions will enable readers to better grasp the findings of my study and contribute to future research in this area.

I would like to extend my heartfelt thanks once again to you and the reviewers for your meticulous review and guidance. It has been an enriching experience, and I am hopeful that my revised manuscript will meet the high standards of your esteemed journal.

Thank you for your time and consideration. I look forward to your feedback.

Best regards,
